# Effectiveness of a pulsed laser in heat-assisted magnetic recording

**DOI:** 10.1038/s41598-023-38398-x

**Published:** 2023-07-16

**Authors:** Yifei Chen, R. H. Victora

**Affiliations:** 1 Department of Physics, University of Minnesota, Minneapolis, MN 55455 USA; 2 Department of Electrical and Computer Engineering, University of Minnesota, Minneapolis, MN 55455 USA

**Keywords:** Electrical and electronic engineering, Information storage, Electronics, photonics and device physics

## Abstract

Recently, much effort has been directed towards increasing the areal density of heat-assisted magnetic recording (HAMR). Here, we use our HAMR recording simulation that employs renormalized media parameters to examine the potential use of a pulsed laser instead of a continuous laser. Proper tuning of the synchronization between magnetic and laser pulses yields improved thermal gradients and comparable (or improved) recording performance relative to a continuous laser. Importantly, it also produces less average heat in the media, which is expected to improve near field transducer lifetime. Results also show that the optimized pulsed laser reduces adjacent track erasure relative to a continuous laser, which is important for non-shingled recording.

## Introduction

Hard disk drives play an important role in our modern lives as the core information storage component of many computers, data centers, etc. According to the prediction of IDC^1^, in the year 2022, people created, saved, and transferred about 97 zettabytes (ZBs) of information annually ($1 ZB = 10^{21}$ bytes) and the size is continuing to increase with a compound annual growth rate of 23%. Global data creation and duplication will rise to 181 ZBs in 2025. The explosive demands for data storage require an increase in HDD capacity as well as areal density (AD)^2–6^.

However, magnetic recording is facing a famous trilemma: recording density, thermal stability, and writability can not be achieved at the same time. To overcome the trilemma, people have proposed HAMR^7–10^. The media is heated by the applied laser to reduce the coercivity, and thus help the switching of magnetization. A laser is focused on the media and locally heats it so that the media temperature is close to the Curie temperature of the recording media. Then during the process of cooling down, a magnetic field is applied, and the information is written. Finally, the information is stored once the temperature is reduced to room temperature^11^.

Conventionally, a continuous laser is used for the heating. The laser is focused on the surface of the media with the help of the near-field transducer (NFT)^12^. During the recording process, the NFT itself will also be heated and the high temperature is a problem for the long-time operation of the NFT, including chemical and mechanical instability. Recently, researchers proposed a pulsed laser to reduce the side effects of heat accumulation^13–17^. This might be helpful because less energy will be generated in the recording system. If similar recording performance could be obtained through short laser-on time, instead of the widely used always-on one, then pulsed laser recording will be advantageous. However, the implementation of pulsed lasers in HAMR faces some challenges. First, data about how the laser pulses affect the recording performance is limited^15^. Also, some researchers found that the SNR would be worse compared to applying a continuous laser^13,17^. Experimentally, other researchers found that a pulsed laser would have a similar performance, but the recording density they used was low^14^. Finally, although one group argued that pulsed laser recording is beneficial, the performance of the comparative continuous laser is poor for unknown reasons^16^.

## Methods

**Figure 1:**
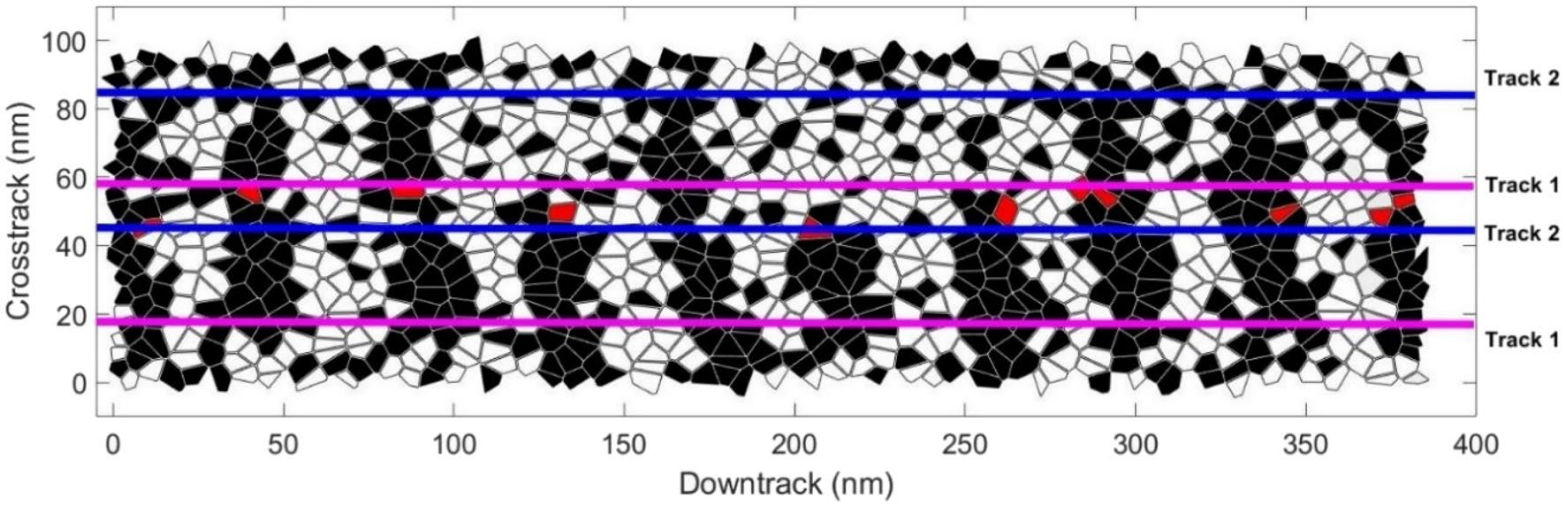
Voronoi grain map of HAMR media. Red grains represent the switched grains after Track2 is written.

This paper aims to optimize and prove the effectiveness of pulsed laser recording. In this work, micromagnetic simulations of the Landau-Lifshitz-Gilbert (LLG) equation are used. The HAMR media is 384 nm along the down-track direction and 96 nm along the cross-track direction. To save computational resources while mimicking the dynamics of magnetic grains, we use the method of renormalization, where the interactions among atomic spins are replaced by larger block spins^18^. Note that each grain exhibits a total spin that can be less than $M_s$ because individual blocks do not align at high temperatures. Each cell has a dimension of 1.5 nm × 1.5 nm × 1.5 nm. Voronoi media^19^ is implemented, and the mean value of grain size is 6.1 nm with a distribution of 19.3%. The non-magnetic boundary is 1 nm, so the grain pitch is 7.1 nm. Two media are included in this work, which are FePt^20^ and exchange coupled composite (ECC)^21,22^. The latter is made up of a 3 nm superparamagnetic write layer ($T_c\sim 900K$) and a 6 nm FePt storage layer ($T_c\sim 700K$). The total thickness for both media is 9 nm. Above the media, we assume that the head moves with a velocity = 20 m/s. The magnetic fly height is 6 nm, the reader width is 25 nm, and the shield-to-shield spacing (SSS) is 21 nm. The default Bit Length (BL) that we used is 20 nm. The recording performance is mainly analyzed through signal-to-noise ratio (SNR) and adjacent track erasure (ATE). Here, SNR is defined as $SNR=10\log _{10}{(signal\ power/noise\ power)}$, which measures the quality of the written signal, while ATE describes how much the written signal will be affected by another signal written in its adjacent track. To simulate the recording process, as shown in Fig. 1, track 1 is first written with 101010... signals. Then, track 2 is written with a pseudorandom signal multiple times. Next, the playback signals of track 1 are obtained based on the reciprocity principle. Finally, we calculate SNR under different write numbers and extract the value of B in the expression $SNR=A+B\times ln(Write\ No.)$, where B represents the magnitude of ATE.

In this paper, we employed the thermal profile of the pulsed laser described in our previous work^23^. In the expression for a pulsed laser given by Eq. 1, x and y stand for the coordinates along down-track and cross-track directions, t is time,$\tau$ represents the decay rate of temperature which is called the time constant, $A^{\prime }$ is used to describe the laser power, v is the head velocity, and $\sigma$ is the standard deviation of the Gaussian function. The decay rate depends on the media structure and its default value is 0.5 ns as suggested by previous work^24^. The full-width half maximum (FWHM) is 30 nm and the laser is assumed to be turned on and off every 0.5ns.

1 $$T({\text{x}},{\text{y}},{\text{t}}) = 300 + \left[ {A^{\prime } \times e^{{\frac{{ - y^{2} }}{{2\sigma ^{2} }}}} \times e^{{ - \frac{{2vt - 2x - \frac{{\sigma ^{2} }}{{v\tau }}}}{{2v\tau }}}} } \right]\left[ {\left( {\frac{{\sqrt \pi }}{2}} \right)\left( {\frac{{\sqrt 2 \sigma }}{v}} \right)} \right]\left[ {{\text{erf}}\left( {\frac{{x + \frac{{\sigma ^{2} }}{{v\tau }}}}{{\sqrt 2 \sigma }}} \right) - {\text{erf}}\left( {\frac{{x - v(0.5ns) + \frac{{\sigma ^{2} }}{{v\tau }}}}{{\sqrt 2 \sigma }}} \right) + {\text{erf}}\left( {\frac{{x - v(1.0ns) + \frac{{\sigma ^{2} }}{{v\tau }}}}{{\sqrt 2 \sigma }}} \right) - {\text{erf}}\left( {\frac{{x - v(1.5ns) + \frac{{\sigma ^{2} }}{{v\tau }}}}{{\sqrt 2 \sigma }}} \right) + \ldots } \right]{\text{ }}$$

## Results

**Figure 2:**
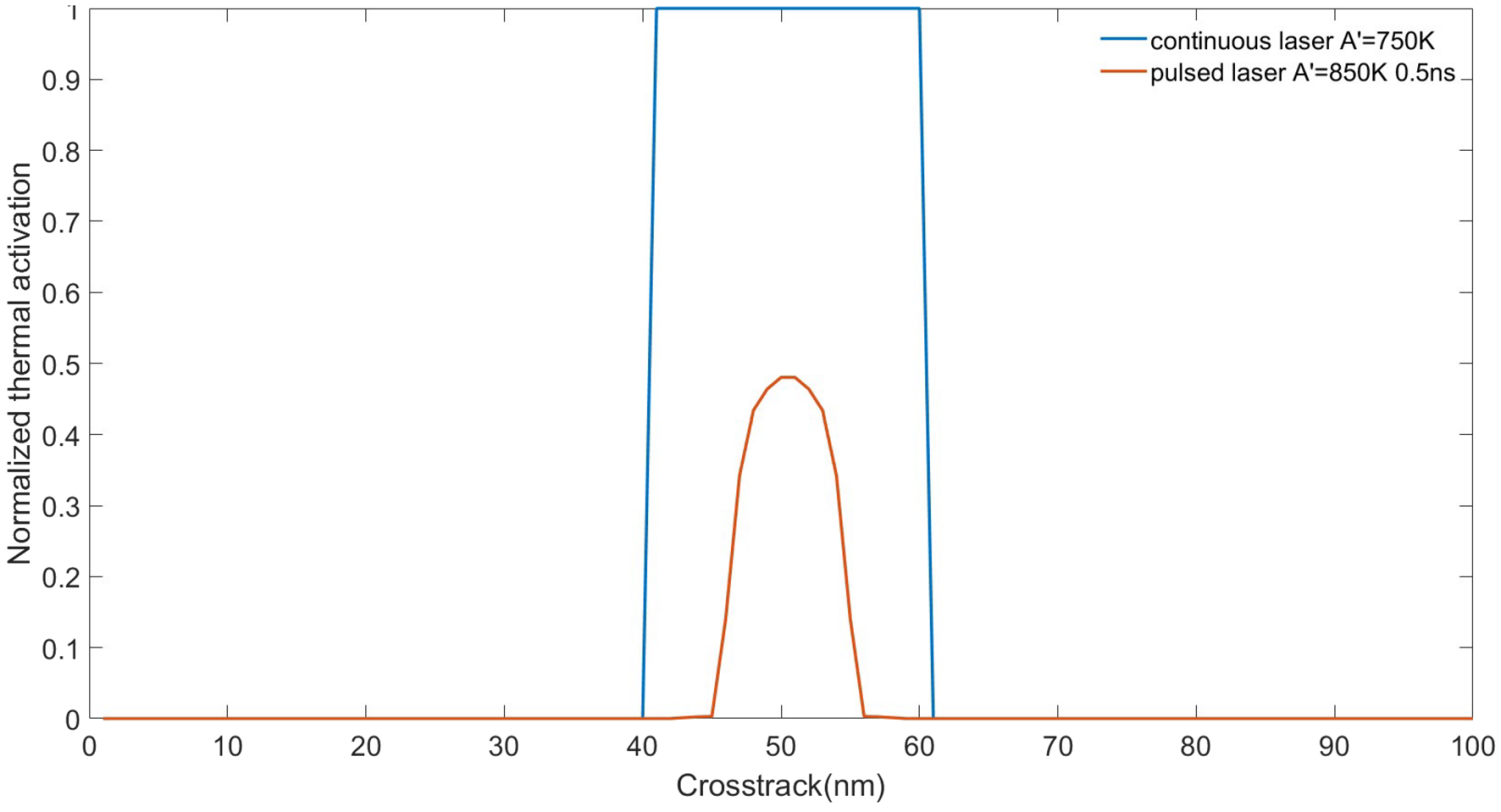
Averaged thermal activation of pulsed laser and continuous laser along the cross-track direction.

We calculated the integrated thermal activation $\int _{0}^{1 n s} e^{-K_{u}[T(t)] V / K_{B} T(t)} d t$ and plotted it along the cross-track direction, which is normalized to the value for the continuous laser, as shown in Fig. 2. It is found that the pulsed laser gives a narrower distribution than the continuous laser, thus the already written track is less easily affected by the signals written in the adjacent track. Then, the boundary of a pulsed laser can be viewed as a narrower track with a high thermal down-track gradient, compared to a continuous laser. Therefore, ATE is expected to be improved by using a pulsed laser.

**Figure 3:**
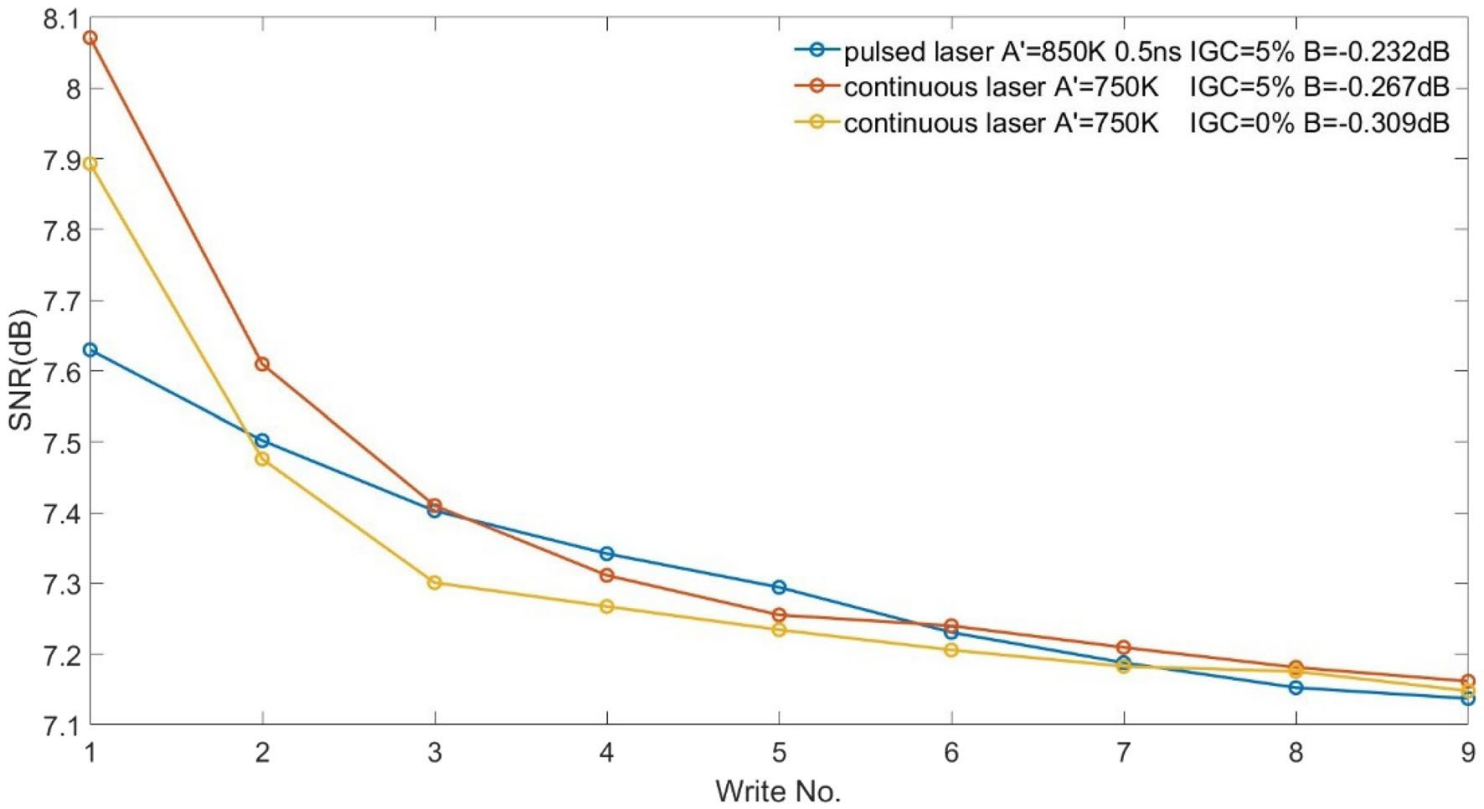
SNR of pulsed laser and continuous laser of FePt media with different values of IGC. B is extracted from $SNR=A+B\times ln(Write\ No.)$.

We introduced intergranular exchange coupling (IGC) for FePt media. The coupling between grains = 5% is found to be optimal for both the continuous and pulsed laser configurations (Fig. 3). Although the SNR of the pulsed laser is lower in the first several writings, it becomes better when the write number is further increased because the pulsed laser has a smaller ATE. By fitting the expression $SNR=A+B\times ln(Write\ No.)$ to the data, the crossovers between pulsed laser and continuous lasers are predicted to happen at write number = 6 and 7, for IGC = 0% and 5% respectively. Also, it is noticed that IGC improves SNR, which could be explained by the bigger bits formed by the interactions between switched and unswitched grains.

**Figure 4:**
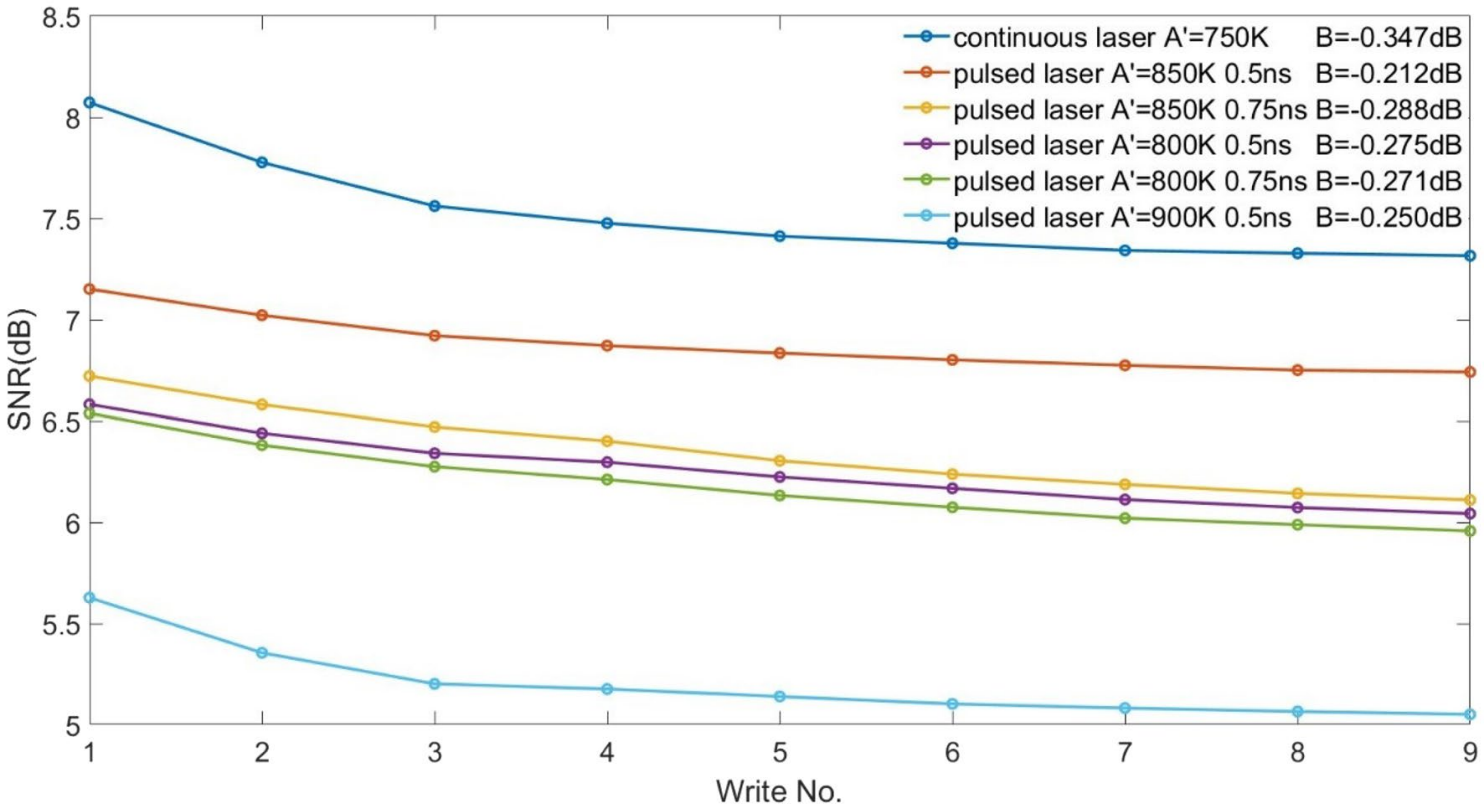
Tuning of SNR using $A^{\prime }$ (800 K,850 K, and 900 K) and time constants (0.5 ns and 0.75 ns) for ECC media.

Next, for ECC media, 10% IGC is applied in the writing layer only and SNRs are calculated under different peak temperatures and time constants. Among the pulsed laser data, the best result is given by peak temperature = 850 K, time constant = 0.5 ns (see Fig. 4). Longer time constants are found to decrease performance, presumably owing to reduced benefit from the pulsing.

**Figure 5:**
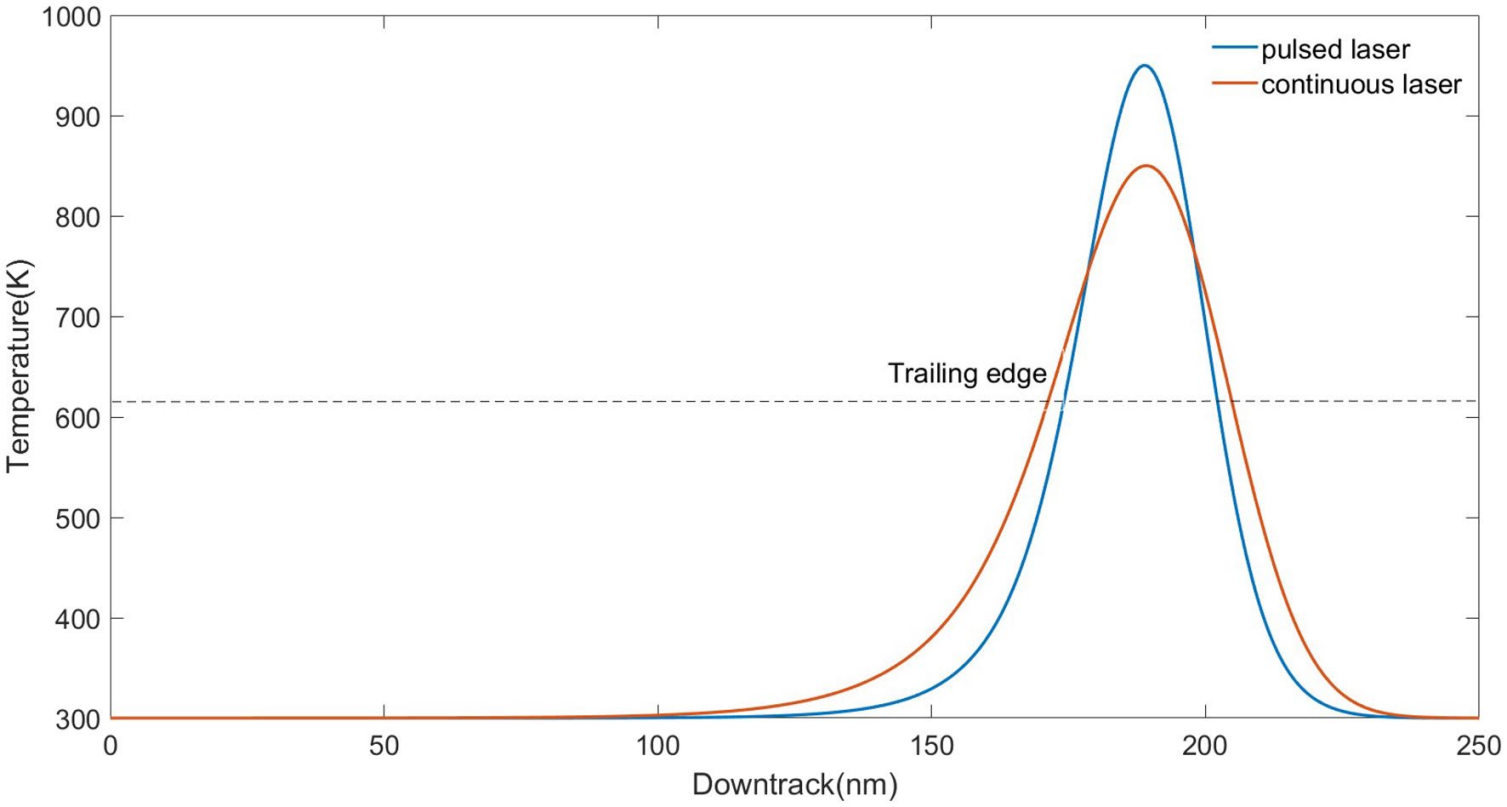
Evaluation of thermal gradient at 625 K (dashed line) for (**a**) continuous laser with $A^{\prime }$ = 750 K, and (**b**) pulsed laser with $A^{\prime }$ = 850 K, time constant = 0.5 ns.

The thermal gradient describes the changing rate of temperature with respect to position, which is an important factor that may affect the recording performance^13,14^. A higher value of thermal gradient at the transition position is desired so that the transition noise could be reduced. So, we calculated the thermal gradient along the down-track direction, for both pulsed laser and continuous laser. The result of normalized temperature against the down-track position is shown in Fig. 5. Here, it is supposed that the head moves with a speed of 20 m/s. The temperature $A^{\prime }$ of the continuous laser is 750 K, while $A^{\prime }$ of the pulsed laser is 850 K, time constant = 0.5 ns. For both cases, the thermal gradient is evaluated at 625 K, in the falling edge of the temperature curve, at t = 9.5 ns and y = 0. We found the pulsed laser has a gradient of 18.2 K/nm, which is higher than the continuous laser (13.0 K/nm). This implies that smaller transition noise should be expected by using the discontinuous laser.

**Figure 6:**
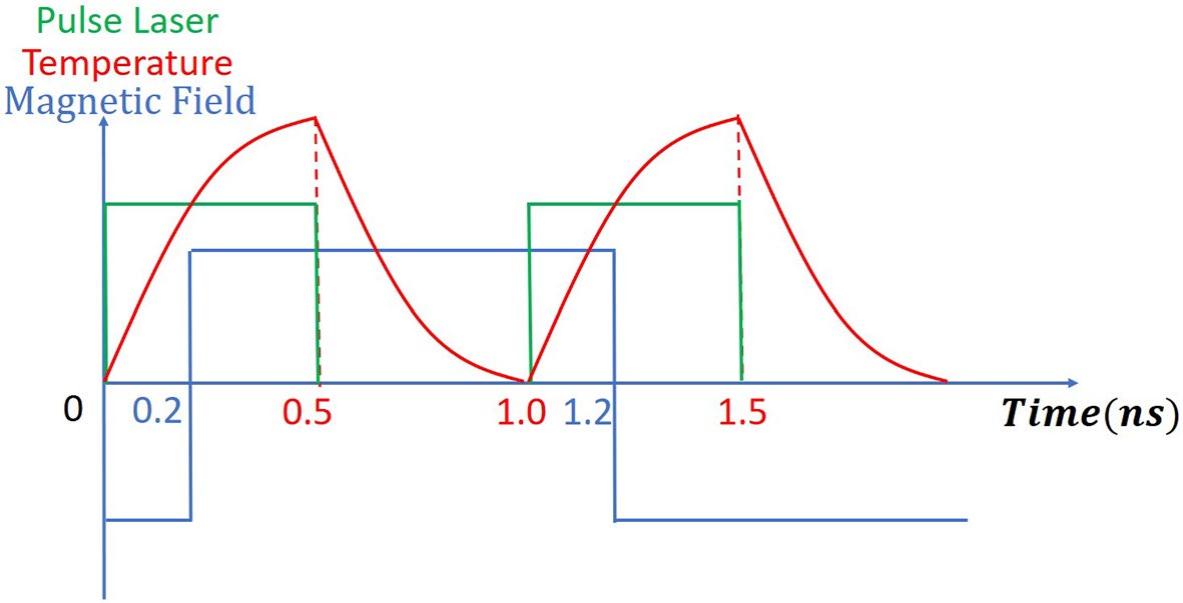
Schematic of pulsed laser, temperature, and magnetic field. Here, the time delay = 0.2 ns, meaning the magnetic field is applied later than the laser by 0.2 ns.

**Figure 7:**
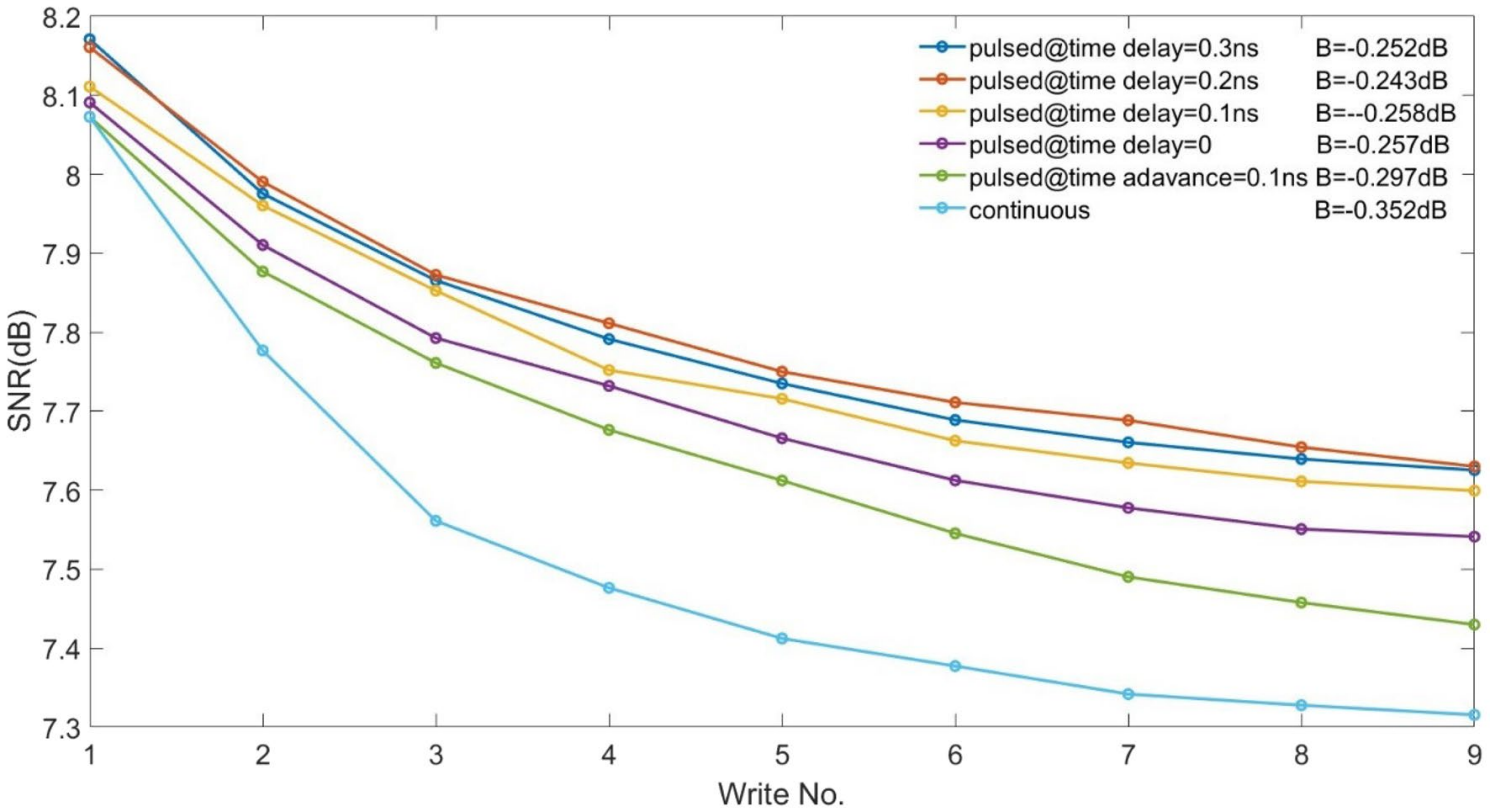
The magnetic fields are turned on using various time delays and advances with respect to the laser pulse, at BL = 20 nm. ECC media, IGC = 10%. Pulsed laser $A^{\prime }$=850 K, continuous laser $A^{\prime }$=750 K.

To synchronize the field and make the transition positions closer to a higher thermal gradient, we delayed (or advanced) the applied magnetic field (see Fig. 6) and calculated the corresponding SNR.which is equivalent to the phase adjustment between laser and magnetic field considered by previous work^14,17^. It is found that with 10% IGC, delaying the turning-on of the magnetic field is beneficial and gives a better SNR than the continuous laser (see Fig. 7). The value of time delay is further optimized and the optimal time delay is found to be 0.2 ns.

**Figure 8:**
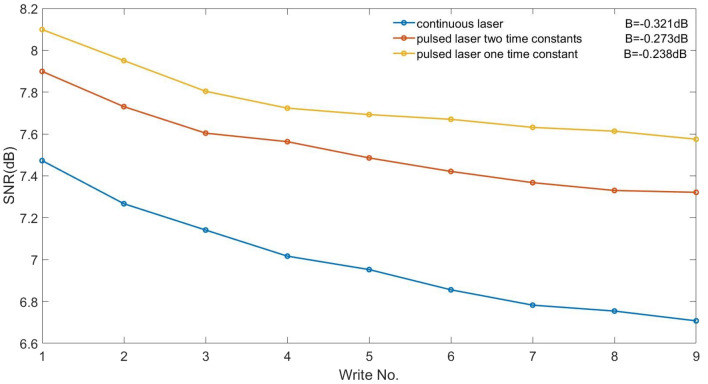
Comparison of the two-time constant model, one-time constant model, and continuous laser. ECC media, IGC = 0%.

Recently, researchers proposed to describe the experimental results using a two-time constant model^25^. To test the impact of this on our results, we assumed a short time constant = 0.5 ns with a weight = 80% and a long time constant = 2.0 ns with a weight = 20%. For the one-time constant model, time constant = 0.5 ns and other parameters are kept at the optimized value. The results, as shown in Fig. 8, imply that the performance of the pulsed laser is still better than the continuous laser, although the superiority is reduced.

## Discussion

In order to further investigate the recording performance, we also calculated Bit Error Rates (BER) and magnetic jitter. Magnetic jitter is calculated as $\sigma _{jitter}=\sqrt{N^{-1}{\textstyle \sum _{i}}(d_i-d_m)^2}$ where $d_i$ is the zero-crossing position of the read-back signal, $d_m$ is the average position and N is the number of transitions^26^. To obtain the value of jitter, 216 transitions from isolated tracks are used for the calculation. The results in Table 1 show that applying a pulsed laser reduces jitter. For comparison, we estimate the jitter from grain size to be 1.13 nm^27^. However, the improvement is more substantial for a short time constant.

**Table 1:** Results of magnetic jitter calculations.

Conditions	Magnetic Jitter (nm)
Continuous laser, $A^{\prime }$ = 750 K, IGC = 0, BL = 20 nm	1.98 ± 0.14
Pulsed laser, $A^{\prime }$ = 850 K, time constant = 0.50 ns, IGC = 0, BL = 20 nm, time delay = 0.2 ns	1.79 ± 0.12
Pulsed laser, $A^{\prime }$ = 850 K, time constant = 0.75 ns, IGC = 0, BL = 20 nm, time delay = 0.1 ns	1.92 ± 0.13

To calculate BER, first, a sampling process is performed on the playback signals, and a 1-D minimum mean square error (MMSE) equalizer is utilized to transform these sampled signals into ideal signals as much as possible. Then, a Viterbi algorithm^28^ is used to decode these equalized signals, and BERs can be obtained by comparing these results with the original PRBS. Here, we used 496 bits for the training of data and 1488 bits for the calculation of BER. The results are displayed in Table 2 and show that the pulsed laser has a similar BER to the continuous one. This agrees with the results of previous research^14^, but with a much shorter bit length(minimum = 10 nm) and thus higher recording density.

**Table 2:** Results of BER calculations with BL = 10 nm.

Conditions	BER	SNR (dB)
Continuous laser, $A^{\prime }$ = 750 K, IGC = 0	0.0047 ± 0.0014	9.9
Pulsed laser, $A^{\prime }$ = 850 K, time constant = 0.50 ns, IGC = 0	0.0047 ± 0.0010	9.6

As a summary, through micromagnetic simulations, we found that the performance of pulsed laser recording could be tuned by peak temperature, thermal time constant, intergranular exchange coupling, and field synchronization. If the parameters are properly selected, applying a pulsed laser in HAMR should lead to a better SNR and ATE compared to a continuous laser. This provides a possible approach to solve the problems of excess heat in a HAMR NFT.

## Data Availability

The datasets generated during the current study are available from the corresponding author upon reasonable request.
